# Quantitative approaches for decoding the specificity of the human T cell repertoire

**DOI:** 10.3389/fimmu.2023.1228873

**Published:** 2023-09-07

**Authors:** Zahra S. Ghoreyshi, Jason T. George

**Affiliations:** ^1^Department of Biomedical Engineering, Texas A&M University, College Station, TX, United States; ^2^Engineering Medicine Program, Texas A&M University, Houston, TX, United States; ^3^Center for Theoretical Biological Physics, Rice University, Houston, TX, United States

**Keywords:** TCR, pMHC, binding prediction, protein-protein interaction, machine learning, deep learning

## Abstract

T cell receptor (TCR)-peptide-major histocompatibility complex (pMHC) interactions play a vital role in initiating immune responses against pathogens, and the specificity of TCRpMHC interactions is crucial for developing optimized therapeutic strategies. The advent of high-throughput immunological and structural evaluation of TCR and pMHC has provided an abundance of data for computational approaches that aim to predict favorable TCR-pMHC interactions. Current models are constructed using information on protein sequence, structures, or a combination of both, and utilize a variety of statistical learning-based approaches for identifying the rules governing specificity. This review examines the current theoretical, computational, and deep learning approaches for identifying TCR-pMHC recognition pairs, placing emphasis on each method’s mathematical approach, predictive performance, and limitations.

## 1 Introduction

The adaptive immune system has the remarkable responsibility of recognizing and eliminating foreign threats, which requires discriminating self from non-self-signatures. T lymphocytes, or T cells, are the cellular mediators of adaptive immunity and accomplish this feat by using their heterodimeric T cell receptors (TCRs). TCRs recognize short peptides bound to and presented by class I and II major histocompatibility complex (MHC) molecules on the cell surface (pMHC). TCR diversity is generated by genetic rearrangement through a V(D)J recombination process (1) capable of generating a staggering diversity of TCRs (estimates range from ∼ 10^20^ to ∼ 10^61^ possible receptors) (2). It is this total diversity together with the relative sparsity of realized samples that complicates the development of inferential modeling procedures capable of predicting TCR-pMHC specificity when test systems differ moderately from training samples (3, 4). Solving this problem would have numerous immunological implications that range from identifying improved antigen vaccines to facilitating optimal selection of adoptive T cell therapy for cancer patients (5, 6).

T cell responses occur when their TCRs bind pMHC with an interaction that ‘appears’ to the T cell as ‘non-self’. In order to avoid detection of abundant self-signatures, T cell precursors (thymocytes) undergo central tolerance to a set of self-signatures via a process called thymic negative selection (7), wherein each thymocyte is exposed to a diverse set of self-antigens, and TCR recognition of any of these self-antigents results in deletion. In addition to central tolerance, a variety of peripheral tolerance mechanisms exist to prevent self-recognition, including T cell anergy, suppression by regulatory T cells (Tregs), and tolerance induction through peripheral antigen exposure (7, 8). Collectively, these mechanisms ensure that mature T cells are selectively responsive to non-self antigens while maintaining a state of immunological self-tolerance.

Owing to the sheer complexity of the adaptive immune response, a number of theoretical and computational models have been explored focusing on various aspects of the problem. These efforts have benefited from the availability of advanced structural characterization techniques, such as X-ray crystallography (9), NMR spectroscopy (10), and cryoelectron microscopy (11), for validation. Moreover, recent advances in high-throughput approaches (12, 13) have significantly increased the available data on which inferential learning-based models can be constructed. Consequently, a number of computational models have been developed to address the need for reliable TCR specificity prediction between a collection of known TCR sequences and putative antigen targets.

**Figure 1:**
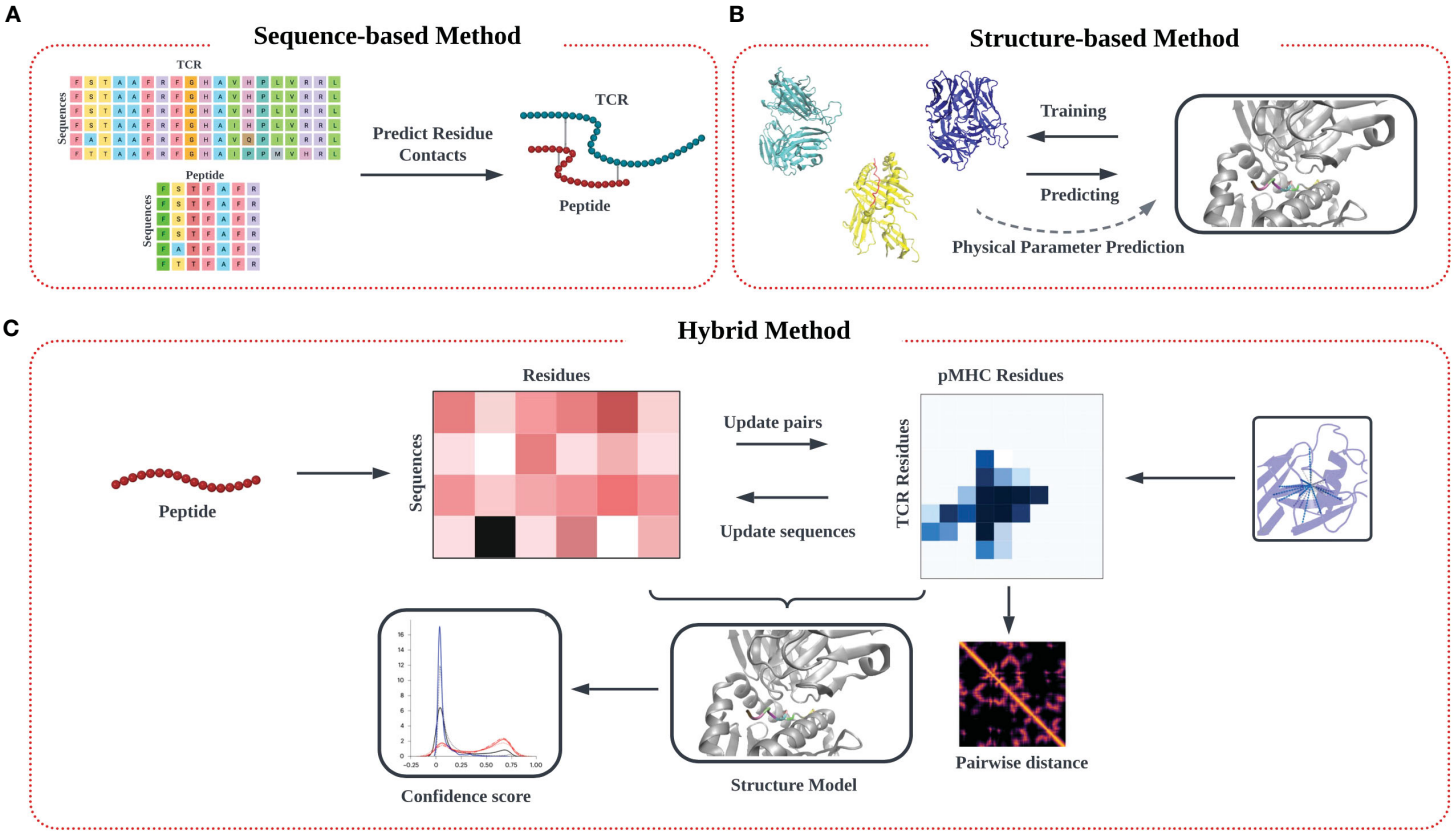
Modeling approach to TCR-pMHC prediction based on input data. **(A)** Models trained purely on TCR and peptide sequence input data feature multiple sequence alignment (MSA) on input data matrices to identify patterns, followed by identification of potential interaction pairs using various algorithms techniques. **(B)** Models trained on input structural data models commonly aim to identify the TCR-pMHC binding interface, along with associated information on binding affinity. Predictions are often made by determining similarity in secondary structure in the interfacial region of the binding interface, from which physical parameters like binding affinity and kinetic data (*K_D_*, *K_off_*) can be estimated. **(C)** A third ‘hybrid’ category of inferential model synergistically combines sequence and structural data in the training step.

In this review, we outline the recent theoretical and computational approaches to TCR-pMHC specificity prediction, emphasizing their strengths and limitations, and offer perspective on the future direction of this exciting modeling effort. In our description of the informatics-based strategies for TCR-pMHC prediction, we discuss current methodology and challenges in four main areas: modeling of TCR-pMHC complex interactions based on (1) sequence-based approaches (**Figure 1A**) (2) structure-based approaches (**Figure 1B**), (3) deep learning approaches, and (4) hybrid approaches (**Figure 1C**).

As we delve deeper into the complex matrix of TCR-pMHC interactions, it becomes essential to illuminate the interplay between specificity and cross-reactivity, two critical factors that significantly shape the predictive modeling landscape. Recognizing the delicate balance between these parameters not only enriches our understanding of various modeling strategies but also refines our approach to interpreting the multi-faceted nature of TCR-pMHC interactions. Cross-reactivity, a fundamental characteristic of TCRs, is an essential consideration in predicting TCR-pMHC interactions. This attribute of TCRs enables them to interact with a myriad of peptide antigens, providing our immune system with its remarkable breadth of response. However, this same characteristic poses a significant challenge in understanding T cell-based therapeutics and similarly in TCR-pMHC specificity prediction. Single TCRs are intrinsically capable of binding to multiple peptide antigens at once, complicating predictions of specificity and off-target effects (14, 15). Traditional prediction methods, which mostly rely on peptide sequence similarities and biochemical similarity, often fail to capture cross-reactivity’s complex nuances. As such, more comprehensive approaches have emerged. For instance, tools like CrossDome (16) aim to predict potential off-target toxicities by leveraging multiomics data from healthy tissues, structural information on TCR-pMHC interactions, and amino acid (AA) biochemical properties. This integrative methodology aids in generating statistically supported predictions that assist in risk assessments and enhance prediction specificity.

## 2 Random energy models of TCR-pMHC interactions

An early quantitative understanding of TCR-pMHC specificity began with the development of random energy models that aimed to explain known properties of the interaction, including TCR specificity versus degeneracy and the potential for self/non-self discrimination. We present here an overview of affinity-driven models, which characterize TCR-pMHC interactions by their free energy of binding. Additional models have considered the effects of kinetic features of the TCR-pMHC interaction (17–19), including on- and off-rates (20), TCR-pMHC binding lifetime (21), and the role of catch vs. slip bonds in TCR activation (22). Both binding affinity and kinetics are likely important for determining the overall outcome of a TCR-pMHC interaction (23–25). Significantly, these approaches can effectively explain the kinetic proofreading aspect of absolute ligand discrimination in a manner that is robust to antigen concentration (24, 26). However, due to the abundance of data on TCR-pMHC binding affinity (via estimated *k_D_* values), we focus our discussion here on affinity-based models.

Early approaches modeled affinity-driven TCR-pMHC interactions using paired strings (27, 28), as detailed in a study by Perelson (29), and review various computational models for receptor representation and properties (30). In these models, interacting TCRs and peptides are represented by AA strings of length *N*. It is assumed that the total TCR-pMHC binding energy can be represented by the sum of individual pairs of interacting AAs:


(1) $$E(t,q)=\sum_{i=1}^{N}J(t_{i},q_{i}).$$

In this case, *E*(*t,q*) is the free energy of interaction between receptor *t* and antigen *q*. *J*(*t_i_,q_i_*) is the interaction energy between the *i*^th^ AAs on the hypervariable (CDR3) region of the TCR (*t_i_*) and the peptide (*q_i_*), respectively, and *N* is the length of the variable regions of the TCR. Using this framework, researchers formulated digit string representations capable of explaining the large degree of alloreactivity observed in post-thymic selection T cell repertoires (31). The initial string model (27) used the set of bounded integers to represent the ‘complementarity’ between AA pairs, *t_i_,q_i_* ∈ {1,2*,…,K*}, with *J*(*t_i_,q_i_*) = |*t_i_* − *q_i_* |, and has been applied to successfully model thymic selection and predict empirically observed T cell alloreactivity rates (27, 28, 32).

Chakraborty and colleagues extended this modeling framework (33) by substituting abstract digit string with experimentally observed AA interactions. This was achieved by replacing *J*(*t_i_,q_i_*) with a pairwise AA potential - chosen to be the Miyazawa–Jernigan energy matrix (34). This modeling framework demonstrated that thymic negative selection favors TCR AAs with moderate interaction strengths to avoid T cell deletion due to high energy interactions with a small set of thymic self-peptide (33, 35). When applied to understand the selective pressures imposed on TCR recognition in the setting of HIV, this framework showed how the peptide binding characteristics of a particular HLA allele restriction resulted in enhanced recognition of viral epitopes (36).

Subsequent modeling efforts have investigated how thymic selection impacts the recognition of tumor-associated antigens using the above framework applied to diagonalized TCR-pMHC interactions (4). Here, the diagonalized interaction assumption simplifies the TCR-pMHC binding interface into a set of one-to-one contacts between the AA residues of the peptide and binding pockets of the TCR. This framework demonstrated that post-selection TCRs may capably recognize single AA differences in point-mutated self-peptides at nearly the same rate as unrelated foreign antigens. This work was then extended to describe the effects of non-diagonal interactions (37), which allow for multiple pairwise TCR-peptide AA contacts. These intricate contacts, identified from the proximity of TCR-peptide AAs in known crystal structures, represented by a TCR-peptide contact map. From this, subtle variations in TCR-peptide binding recognition profiles manifest in variable weights of interaction assigned to each of the peptide AA positions. An extension to (33) considered non-uniformity of these weights (37). The contact map *W* = (*W_ij_*) contains weights *W_ij_* for interactions between *t* and *q* in a given structure, and an associated AA interaction coefficient,


(2) $$E(t,q)=\sum_{i,j}W_{ij}J(t_{i},q_{j})$$

**Figure 2:**
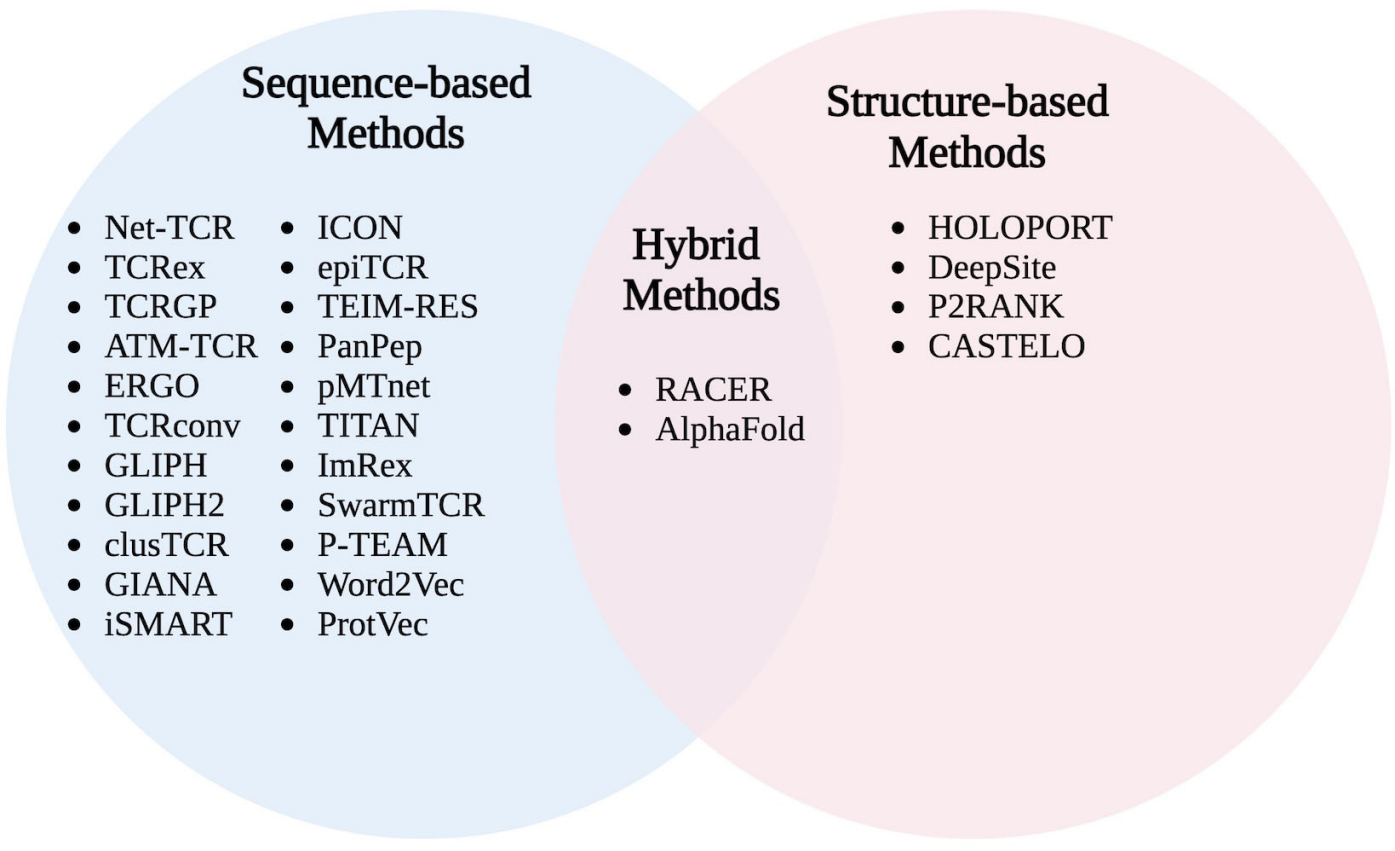
List of commonly used inference-based models of TCR-pMHC specificity partitioned by approach: sequence-based, structure-based, and hybrid.

Using this framework, non-uniformities in contact maps, which are highly variable for given MHC allele variants, can result in high-contacting peptide AA positions. At these positions, single-amino acid changes in wild-type peptides, such as cancer neoantigens or single nucleotide polymorphism peptide variants, result in an enhanced difference in the binding interaction that may ultimately enhance or break immunogenicity. Intriguingly, these statistical models predict a high likelihood of point-mutated self-peptide recognition, which suggests that thymic selection is more akin to a T cell memorization task directed at a list of important self-antigens to avoid rather than an intricately curated list of self-peptides whose tolerance confers wider immune protection. In order to improve forecasting capabilities and reduce the experimental efforts required to search for meaningful TCR-pMHC pairs, advanced machine learning algorithms have been incorporated into biophysical and probabilistic models to create data-driven and trainable predictive models. **Figure 2** provides a non-exhaustive summary of recent open-source computational methods.

## 3 TCR-pMHC specificity prediction methods

The TCR-pMHC specificity prediction methods leverage advanced computational techniques to predict the highly specific interactions between TCRs and pMHC that are crucial to the initiation and effectiveness of adaptive immune responses. The methods discussed in this review can be classified into sequence-based, structure-based, and hybrid models. Each category uses different strategies to evaluate TCR-pMHC specificity, with sequence-based models relying on sequence similarities, structure-based models using three-dimensional structural information, and hybrid models combining these strategies. Various models have been developed to accomplish this, and their performance was assessed based on model generalization and robustness. However, the study found that these models struggle with generalization to peptides not seen in the training data and that their performance fluctuates depending on data size and balance, indicating that predicting TCR-pMHC binding specificity remains a significant challenge. Thus, additional high-quality data and innovative algorithmic approaches are necessary for further advancements.

### 3.1 Sequence-based approaches

Arguably the greatest challenge in predicting TCR specificity arises from the diversity of possible TCR and peptide combinations relative to those that can be studied or even realized in a single individual (38, 39). To experimentally identify relevant TCR-pMHC pairs, pMHC tetramers are often used to experimentally identify TCRs that interact with sufficient binding affinity. The affinity-based screening of TCRs can be done in a high-throughput manner (40). In addition to theoretical modeling, inferential statistical learning provides a complementary approach for studying this problem by imputing known (non-) examples of favored TCR-peptide interactions interactions. These computational models (**Figure 1A**) can be distinguished based on whether or not previously identified TCR-pMHC interactions are used in training, which are given by supervised and unsupervised learning approaches, respectively.

#### 3.1.1 Supervised learning

Sequence-based prediction models refer to machine learning algorithms designed to learn a predictive function that identifies informative features and from them accurately predicts the cognate epitope of an input T cell receptor (TCR) with unknown specificity. Features are learned based on a set of known examples and non-examples of TCR-peptide pairs provided in a training dataset. Capietto et al. (41) demonstrated that peptide mutation positions matter in neoantigen prediction pipelines, and the use of this feature led to improved neoantigen ranking. Other studies found that immunogenic peptides have more hydrophobic AAs at TCR interaction sites and that AAs molecular weight, size, and charge are useful for TCR-pMHC complexes (42–44).Numerous computational tools now exist that use known TCR and peptide sequences to predict TCR-epitope interactions by binary classification, including such as NetTCR (45), TCRex (46), TCRGP (47), and ATM-TCR (48). Furthermore, there has been a progression towards developing TCR-epitope binding prediction models that are not limited to specific peptides, such as SwarmTCR (49), ERGO (50), pMTnet (51), ImRex (52), TITAN (53), and TCRconv (54) which instead utilize known binding TCRs to train the models. While these models work well with peptides having an abundance of known TCR interactions, they often struggle in predicting behavior for peptides having few known interactions or those not included in the training data, attributed to the large diversity of the interaction space. Many approaches consider TCRs and peptides as linear sequences of AAs, while others include a description of their three-dimensional orientations during the TCR-pMHC interaction.

Each of the above cases utilizes different machine learning procedures to achieve a variety of descriptions of TCR-pMHC specificity. NetTCR implements convolutional neural networks (CNN) (55, 56) in conjunction with multiple dense layers (Section 4) to learn the interactions between TCRs and epitopes associated with the most common human MHC allele variant, HLA-A*02:01. This method faces limitations due to the vastness of the TCR-pMHC space and insufficient experimental training data, which challenge current computational algorithms. ImRex, and TITAN are state-of-the-art TCR-epitope binding prediction models utilizing CNNs. ImRex, inspired by image processing CNNs, transforms CDR3 and epitope sequences into interaction maps. It considers the pairwise differences of selected physicochemical properties of the AAs in the sequences, making the maps interpretable as multi-channel images, and predicts TCRepitope binding through a multi-layer CNN. ImRex’s strength rests in its ability to recognize TCR-specific epitopes from unseen sequences that resemble the training data, improving its generalization performance for TCRepitope recognition. On the other hand, TITAN employs a one-dimensional CNN with a contextual attention mechanism (Section 4). It separately feeds encoded CDR3 and epitope sequences into convolutional layers, uses context attention layers for each, and concatenates attention weights. Significantly, TITAN extends beyond simple encoding by employing SMILES sequences for epitopes at the atomic level. In combination with transfer learning, this sophisticated method expands the input data space and enhances model performance. TITAN’s attention heatmaps provide insights into biological patterns and suggest that data scarcity in epitopes can implicitly treat them as distinct classes, which could impact unseen epitope performance in complex models. Both models apply feature attribution extraction methods to explore underlying biological patterns.

TCRex utilizes a series of decision trees (57), which combines with classifiers and ensemble regression trees, to build an epitope-specific prediction model. While this method has been successfully applied for data classification and regression, several challenges involve a lack of generalizability to non-HLA A*02:01 cases and susceptibility to overfitting. EpiTCR (58), which uses the Random Forest algorithm, also integrates several crucial elements for increased precision, can significantly mitigate these issues. These include sequence encoding based on BLOSUM62, zero-padding to maintain sequence uniformity, and utilization of peptide-presenting MHC data in the predictions, providing a comprehensive approach to TCR-peptide specificity prediction. This is done by utilizing a large dataset from various public databases (over 3 million), which are encoded by a flattened BLOSUM62 matrix, and is known for their high sensitivity and specificity in detecting such interactions.

Transitioning from this approach, another promising methodology is presented by the Predicting T cell Epitope-specific Activation against Mutant versions (P-TEAM) model (59). P-TEAM, a Random Forest-based model, proficiently predicts the effect of epitope point mutations on T cell functionality. It provides quantitative predictions for altered peptide ligands and unseen TCRs, showcasing high performance and potential applicability in immunotherapy development. Several bioinformatic approaches, including SwarmTCR, predict antigen specificity from sequences. These tools optimize CDRs to enhance TCR specificity predictions using labeled sequence data. With robust performance on both single-cell and bulk sequencing data, it offers biologically interpretable weights, providing crucial insights into immune responses related to various conditions. TCRconv, a state-of-the-art deep learning model, is designed for predicting TCRs and epitope interactions. Using a protein language model and convolutional networks, it identifies contextualized motifs, improving the accuracy of TCR-epitope predictions. TCRconv, applied to COVID-19 patients’ TCR repertoires, provides enhanced understanding of T cell dynamics and disease phenotypes, highlighting potential applications in infectious diseases, autoimmunity, and tumor immunology. TCRGP relies on analyzing both *α* and *β* chains of the T cell receptor (TCR) in a Gaussian process method in order to determine which CDRs are crucial for epitope recognition. ERGO incorporates Long Short-Term Memory (LSTM) (60) and autoencoder (AE) (61) models (Section 4) on a variety of inputs, including the TCR*αβ* sequences and the VJ genes for each TCR (50). Similarly, ATM-TCR predicts the affinity between TCR and epitope based on a computational model. According to this model, AA residues within TCR and epitope sequences are considered in the context of how they interact with each other using a multi-head self-attention network (Section 4) (62). The TCR-pMHC binding prediction network (pMTnet) approach is a computational model that applies transfer learning (63) (Section 4), a form of deep learning that leverages knowledge gained from prior tasks, to predict TCR binding specificities for neoantigens and other antigens presented by class I MHCs. This model is directed at addressing the challenge of predicting TCR-antigen pairing and has demonstrated significant advancements over previous methods. As validated through the characterization of TCR-pMHC interactions in human tumors, pMTnet provides superior predictive accuracy. It effectively distinguishes between neoantigens and self-antigens, evaluates TCR binding affinities, and calculates a neoantigen immunogenicity effectiveness score (NIES). This ability allows for a comprehensive analysis of tumor neoantigens’ role in tumor progression and immunotherapy treatment response, emphasizing the method’s important contribution to understanding immunogenic tumor antigens and their relationship to T cell proliferation. On the other hand, pMTnet exhibits inferior performance in the few-shot settings and fails to recognize TCR binding to novel peptides in zero-shot settings, despite their robust performance in settings with an ample volume of known TCR binding data. Conversely, the effective implementation of the TITAN model, trained specifically with COVID-19 data using peptides with sparse known binding TCRs, is limited by the breadth of available empirical data, thus posing a significant hurdle to the prediction of TCR interactions with new or rare peptides.

Another perspective by Meysman et al. (64) compares TCR-pMHC binding approaches and concludes by stressing the need for an independent benchmark. The authors find improvements in predictions accuracy when including CDR1/2 information, but leave open a complete investigation of the impact of data imbalance with respect to the biological context of included training examples, size, and overtraining on model performance. The lack of standardization for these factors complicates TCR-pMHC binding prediction and comparative benchmarks. Subsequent evaluations by Meysman’s group underscored the unpredictability of unseen epitope predictions, reinforcing the call for advanced models and rigorous, standardized evaluation protocols (65).

In this application, T cell receptor (TCR) sequences are transformed into numerical representations through a process called encoding. Encoding methodologies commonly utilize physicochemical properties or one-hot encoding, which is a technique where each unique AA in a sequence is represented by its own unique binary code, making each one distinct for computational models. The immuneML (66) platform extends the capabilities of earlier methods like DeepRC (67), GLIPH2 (68), and TCRdist (3) to train and evaluate machine learning classifiers at the receptor level, and it accomplishes this by incorporating a variety of encoding methods for sequence data, including *k*-mer frequency decomposition, one-hot encoding, disease-associated sequence encodings, and repertoire distance encodings, facilitating comprehensive sequence analyses. This platform offers a variety of models, encompassing K-Nearest Neighbours (KNN), logistic regression, random forests, and the TCRdist classifier, among others, providing a versatile toolkit for receptor analysis. The inclusion of the TCRdist classifier allows for meaningful distance measurements between receptors, taking into account the unique characteristics of TCRs, like their exceptional variability and adaptability in recognizing different antigens. These models, therefore, provide a versatile toolkit for receptor analysis. The enhanced reproducibility, transparency, and interoperability offered by ImmuneML effectively overcomes traditional challenges in Adaptive Immune Receptor Repertoires (AIRR) machine learning. The review (69) provides a comprehensive overview of other advanced methods and computational tools emerging in this area of research, which facilitate a more complete and nuanced understanding of T cell receptor sequences and their functional implications. These include V(D)J recombination (70), single cell sequencing (71), multimodal experiments (72), flow cytometry (73), mass cytometry (CyTOF) (74), RNA sequencing, feature barcoding, and cell hashing.

A persistent challenge for these approaches is that such models often make incorrect predictions because of limited validated pMHC-TCR interaction data (38, 39, 75). According to Deng et al.’s study, the effectiveness of these models is significantly affected by the data balance and size (75). Furthermore, the models exhibited limitations in generalizing to untrained peptides, emphasizing the need for improved data collection and algorithmic improvements. Similarly, epitope binding affinity models such as TCRGP and TCRex cannot be used to investigate novel or understudied systems since they require that a new model be constructed for each epitope once there are a sufficient number of identified cognate TCRs. NetTCR, ERGO, and ATM-TCR models are all capable of predicting novel or rare epitopes, but they perform poorly overall as evaluated by the area under the receiver-operating characteristic curve (ROC-AUC) metric. A promising approach that addresses this issue is the Pan-Peptide Meta Learning (PanPep) (76), a meta-learning method combined with a Neural Turing Machine (NTM) (77). Meta-learning allows for the model to learn from a set of tasks and then apply insights gained to predict binding specificity for new and unknown tasks, such as predicting binding specificity for neoantigens or exogenous peptides. Using a NTM adds external memory to the system, ensuring retention of learned tasks and thereby improving prediction accuracy for TCR binding specificity to unknown peptides. Despite meta-learning’s effectiveness when there are few examples available, its reliance on labeled data can limit its application. A powerful alternative to manual labeling, unsupervised learning is capable of extracting meaningful patterns from unlabeled data.

#### 3.1.2 Unsupervised learning

In contrast to supervised learning, unsupervised learning does not rely on the availability of known TCR-peptide pairs, instead learning to group TCR, antigen, or HLA inputs based on statistical variation inherent in their sequences. Consequently, a number of approaches have attempted to train unsupervised models. The GLIPH (Grouping Lymphocyte Interactions by Paratope Hotspots) method utilizes high throughput data analysis to identify distinct TCR sequences that recognize the same antigen based on motifs shared in their CDR3 sequences (78), has a significant place in TCR-antigen interaction studies, and enhances TCR specificity prediction when combined with other resources, such as the V(D)J database. This clustering assists in pinpointing known TCR specificities. It’s crucial, though, to understand the inherent constraints of GLIPH, including challenges in managing large datasets and the model’s reliance on other resources for direct antigen interaction predictions. Understanding the specificity of the T cell repertoire in this context requires the identification of related systems from a small training subset in a high-dimensional space.

Given the absence of *a priori* identified specificity groups, to clustering methods may outperform traditional supervised classification schemes. In comparison to randomly grouped clones, TCRs within the cluster exhibited highly correlated gene expression and shared a common specificity. TCRs are clustered by GLIPH based on two similarity indexes: 1) global similarity, which refers to the difference between CDR3 sequences up to one AA, and 2) local similarity, which refers to the fact that two TCRs share a common CDR3 motif of 2, 3, or 4 AAs (enriched relative to that of a random subsampling of unselected repertoires). Moreover, the GLIPH algorithm, by adeptly recognizing shared motifs within the CDR3 of TCR sequences, has a significant place in TCR-antigen interaction studies and enhances TCR specificity prediction when combined with other resources, such as the V(D)J database. This combination assists in pinpointing known TCR specificities. It’s crucial, though, to understand the inherent constraints of GLIPH, including challenges in managing large datasets and its reliance on other resources for direct antigen interaction predictions

TCR sequences with a shared epitope specificity carry motifs that are statistically enriched in the peptides they mutually recognize. A method that builds on GLIPH to include motif-based clustering (GLIPH2) (68) is fast but lacks specificity, while clusTCR (79) is faster because it encodes CDR3 sequences using physiochemical features by representing them as integers with an assigned Hamming distance. This comes with the tradeoff of lacking TCR variable gene information, and thus clusTCR has lower clustering purity defined as the proportion of items in a cluster that belong to the most common category or group. To address this challenge, the Geometric Isometry-based TCR Alignment Algorithm (GIANA) (80) transforms CDR3 sequences using the Nearest Neighbor (NN) search in high-dimensional Euclidean space to solve the problem of sequence alignment and clustering. In these methods, similar features are found among TCRs recognizing the same target. Similar TCRs can be grouped/clustered by predicting which targets they will recognize in this way. Various additional factors, including alignment of T cell receptors and identification of TCR-antigen interactions using high-throughput pMHC binding data, are also considered in other methods (13, 81).

The identification of Tumor-Associated Antigen (TAA)-TCR specificity as a subset of the overall predictive task has historically been challenging in large part owing to the fact that a majority of TAAs are point-mutated self-peptide. Given the tremendous clinical value of reliably predicting TCR-TAA specificity, several algorithms have been developed for this specifically. In one case, called TCR Repertoire Utilities for Solid Tumors (TRUST), assembles hypervariable CDR3 regions of TCRs, and then applies a clustering method called immuno-Similarity Measurement using Alignments of Receptors of T cells (iSMART) to group TCRs based on their antigen specificity (81). Despite these advances, no systematic evaluation of these methods has been conducted on large and noisy datasets, and experiments to reduce nonspecific multimer binding, validate correct folding, and improve signal-to-noise ratios are still required. Integrated COntext-specific Normalization (ICON) (13) is a notable development in this field that identifies TCRantigen interactions in high-throughput pMHC binding experiments. The experimental approach consists of initial filtering of T cells based on single-cell RNA-seq, followed by background noise estimation via single-cell dCODE-Dextramer-seq, and then lastly TCR identification via paired *αβ* single cell TCR sequencing. The TCRAI neural network predicts and characterizes these interactions and in doing so reveals conserved motifs and binding mechanisms. The combination of ICON and TCRAI leads to the discovery of novel subgroups of TCRs that interact with a given pMHC via diverse mechanisms.

Although many clustering-based approaches have been developed, conventional clustering methods usually perform poorly on high-dimensional data often as a result of inefficiencies in the defined similarity measures (82–84). On large-scale datasets required for studying TCR-pMHC specificity, these methods are generally computationally formidable. Consequently, raw data is often mapped into a more suitable feature space where existing classifiers can separate the generated data more easily, followed by dimensionality reduction and feature transformation. A number of existing transformation methods have been applied to this problem, including linear methods like Principal Component Analysis (PCA) (13) as well as non-linear strategies such as kernel methods and spectral methods (85). Clustering methods often encounter difficulties when dealing with complex structures owing to the fact that their clustering criteria are based on simplified criteria, in contrast to Feed-Forward neural networks and Deep Neural Networks (DNNs), that provide highly non-linear transformations of data that can be used to cluster the data. Further advancements in artificial intelligence have led to deep learning surpassing other statistical methods in many domains (86–91). Unlike traditional machine learning algorithms, which often struggle to assimilate complex features from data, the success of deep learning relies on understanding and interpreting data, which occurs by first learning simple patterns at initial levels of the algorithm and complex patterns at higher ones (92).

Several developed approaches utilize deep learning in an unsupervised manner, and although we discuss the specifics of the deep learning algorithms in Section 4, we will touch briefly on several here. One area where unsupervised deep learning applied to identify meaningful sequences includes the application of Natural Language Processing (NLP) algorithms based on word embedding, such as Word2Vec (93) and ProtVec (94). These algorithms offer a novel approach to understanding the relationship between TCR sequences and antigen binding. By leveraging the concept of word embedding from NLP, they are capable of capturing semantic or functional similarities among TCR sequences, much like similar words in a language (95). Therefore, if two TCR sequences share common motifs, it suggests they may bind to similar antigens. Consequently, these algorithms are valuable tools in immunoformatics, converting raw TCR sequence data into a format conducive to modeling and predicting TCR-antigen interactions. Word2Vec interprets non-overlapping 3-mer sequences of AAs, while ProtVec represents proteins as the sum of overlapping sequence fragments of length *k*. These approaches had several limitations, including limited interpretability due to the lack of biophysical meaning of three-residue segments of protein sequences, and overlapping models often do not out-perform non-overlapping models (94). Recurrent Neural Networks(RNN) (96) were proposed to improve these initial schemes. The RNN model is a sequence-based representation method averaging over the representations of each residue to produce a fixed-length real representation of arbitrary-length protein sequences. This scheme is further improved by implementing a transformer, which differs from RNNs by its incorporation of parallel task assignment. Models based on the transformer were found to be superior to traditional LSTM-based approaches (a variety of RNNs introduced in Section 3.1 and discussed further in Section 4) (60) when applied to tasks such as TCR-pMHC interactions, protein docking, and protein structure prediction, since in these cases the RNN model struggles to capture long-range relationships and does not include parallelizability (97, 98).

More recently, AlphaFold, an artificial intelligence system developed by DeepMind that predicts protein structure using primary sequence information, has been applied to the TCR-pMHC specificity problem (99). This method is a transformer model that utilizes an attention mechanism in order to operate within each row of a Multiple Sequence Alignment (MSA), which generally the alignment of multiple protein sequences of similar length to maximize the positional correspondence of homologous residues across these sequences. This attention mechanism (100) allows the model to focus on specific parts of the sequence, providing a more comprehensive understanding of the relationship between residues and protein folding. The ultimate output is in the form of an accurate 3-dimensional structure that can be assessed for binding specificity. We note that because of this, AlphaFold is a pure sequenced-based prediction model since no structural data is used as input (101).

Lastly, AEs and Variational Autoencoders (VAEs) (102), which stochastically map the input space to the latent space, have surpassed former techniques in the field of sequence-based representation. In contrast, the VAE model is designed to capture the dynamics of the peptide-MHC binding process and to identify per-residue binding contributions by providing a stochastic map between the input and latent space. VAEs in peptide-MHC binding optimization have great potential for advancing the design of vaccines and immunotherapies (103). One recent study, TCR–Epitope Interaction Modelling at Residue Level (TEIM-Res) (104), uses the sequences of TCRs and epitopes as inputs to predict pairwise residue distances and contact sites. An epitope feature vector generated by an AE is fed into an interaction extractor for global epitope information. Using this approach, the method was able to predict TCR-epitope interactions at the residue level, outperforming existing models and demonstrating versatility in mutation and binding pattern analyses.

In addition to supervised and unsupervised learning methodologies, negative data plays a crucial role in enhancing model accuracy and preventing overfitting. By providing contrasting data, negative data aids in identifying patterns and trends in positive data, leading to a more enriched learning process (105). However, while useful for TCR–epitope binding prediction, this study also uncovers the potential pitfalls of its application. The bias it introduces can lead to a dip in model performance in practical scenarios. For instance, the PanPep model was observed to underperform with shuffled negative data. As a result, it is imperative to seek more effective strategies to preserve model practicality while also enhancing applicability, including the uniform employment of a negative sampling strategy during both the training and testing phases (106).

### 3.2 Structure-based approaches

Whereas sequence-based approaches contain no explicit spatial information on the interacting system, several alternative strategies have leveraged structural knowledge of the TCR-pMHC interaction to aid in understanding specificity. When available, structural templates couple primary sequence data with significant spatial information of the interacting pairs, thereby enabling sophisticated computational methods for representing and analyzing structures (**Figure 1B**). Additionally, two models have been developed to explain the T cell’s ability to discriminate between self and non-self pMHCs that utilize the identification of a specific conformational change in the TCR complex and kinetic thresholding (23–25). Direct measurements of signaling molecules and pMHC-TCR ligand interactions are used to develop a model that accounts for the characteristics of T cell signaling in response to antigens.

Despite a large abundance of protein crystal structures (currently over one million in the Protein Data Bank), the number of identified TCR-pMHC crystal structures is quite limited (on the order of hundreds of TCR-pMHC complexes), likely due to the difficulty in producing these complexes in large quantities and in conditions suitable for crystallization. Computational methods for structure representation and analysis include Molecular Dynamics (MD) simulations, homology modeling (107), machine learning, alchemical free energy perturbation (108), and hybrid approaches.

MD simulations have also been used to establish a detailed, all-atom description to better understand TCR-pMHC specificity (109, 110). MD analysis provides an in-depth, mechanistic understanding of TCR and pMHC interactions. However, due to the high computational cost of these approaches, an MD-derived understanding of TCR-pMHC specificity is at present restricted to a small collection of TCRs and peptides in a given analysis. Nonetheless, these insights are critical to predicting TCR-pMHC specificity, as they allow for an understanding of the molecular behaviors and relationships that underpin this complex biological interaction. In this way, MD simulations effectively bridge the gap between fundamental biophysical interactions and the computational prediction of TCR-pMHC binding. This approach begins by generating an initial structure, which can be achieved through side-chain substitution, homology modeling (107), and ligand-protein docking (111), and proceeds using time-dependent simulations of atomic motions in the system, MD simulations account for both the main-chain conformational flexibility and the solvation and entropy effects. The simulation protocols themselves can be accelerated through the use of coarsegraining, increased masses (112), virtual sites (113), n-bead models (114), or the movement of rigid protein regions (115). A variety of pertinent features, including RMSD, RMSF, Solvent-Accessible (SASA), PCA, and hydrogen bonds can be analyzed based on MD simulations, and geometric approaches (116) have also been developed to analyze the binding orientation between the heavy and light chains of antibodies and the TCR *α* and *β* chains. Collectively, this approach can provide highly detailed information on the dynamics of TCR-pMHC systems. However, the high computational cost of performing full MD simulations limits feasible analyses to several TCR-pMHC pairs (117).

Molecular Mechanics (MM) provides a complementary approach to study the bound TCR-pMHC complex using molecular docking techniques. The molecular docking process has two key applications: binding mode prediction and virtual screening. The former involves optimizing the 3D conformation of a peptide when it binds to its target receptor, while the latter entails evaluating a vast number of potential peptides to identify those that can bind to the target receptor (118). In studying the TCR-pMHC interaction, both MD and MM approaches are both challenged by cases having significant peptide flexibility, since a peptide with more flexible bonds can adopt more conformations. In addition to the position and orientation of the peptide inside the receptor’s binding cleft, docking methods must consider these alternative conformations in order to determine the most suitable binding mode.

The field of MM utilizes simulation-based prediction methods, which involve tracking the time evolution of a molecular system through the use of an energy potential. The quality of the potential, or score function, plays a crucial role in protein structural modeling, as it describes the potential energy landscape of a protein. Score functions may also contain knowledge-based terms to distinguish native from non-native conformations. MD or Monte Carlo (MC) simulations with advanced force fields or score functions can accurately reproduce the statistical behavior of biomolecules. The MM-based task of learning a force field with predictive utility has recently been augmented by incorporating deep learning-based approaches. These approaches represent each atom’s chemical environment through graph convolutions (119) and by doing so aim to enhance the accuracy and reliability of MM predictions, through the capture of complex atomistic relationships in local and global chemical environments and generation of transferable, interpretable features that facilitate end-to-end learning. These approaches can broadly be categorized into two categories: graph-based and fingerprint-based.

Graph-based approaches construct a mathematical graph of molecules, containing atoms as nodes and chemical bonds as edges. They maintain structural and chemical information and preserve topological complexity to facilitate more detailed and complex molecular structural analyses, which can be used to predict chemical reactions and molecular docking. In contrast, fingerprint-based approaches represent molecules as binary digits. While these methods provide a computationally efficient, fixed-length representation, they simplify the molecular structure and may lose fine-grain detail about the exact structure and topology. Dual methods that combine both strategies also exist and have been applied to studying the TCR-pMHC interaction. Collectively, these approaches have been shown to enhance the accuracy of molecular modeling in describing simple molecular pairs and possess potential for describing more complex biological processes, including protein complex interactions. The current methodology for computational Protein-Protein Interaction (PPI) prediction is largely based on deep learning methods.

One example of a dual methodology is a multiscale graph construction of HOLOPROT (120), which connects surface to structure and sequence, demonstrates the utility of hierarchical representations for binding and function prediction. Using geometric deep learning and mesh CNN (55, 56) embed protein surface patches into fingerprints for fast scanning and binding site identification, eliminating the need for hand-crafted or expensive pre-computed features. Importantly, these methods do not perform structural blind docking, which involves determining the binding site, orientation, and location of the two molecules, and internal conformational deformations during binding. Consequently, they capture and predict molecular interactions based on effective molecular representations and efficient learning algorithms, without explicitly simulating binding dynamics.

Another example includes Graph Deep Learning (GDL) methods. While they are reliant on known structural data, GDL approaches offer unique advantages in capturing the complex, non-linear relationships between features, making them potentially valuable for predicting protein structures (121), interactions (122), and functions (123). AlphaFold has revolutionized PPIs modeling with its sophisticated end-to-end approach, which outperforms traditional docking methods. In order to accurately model complex interactions, such as T cell receptor-antigen complexes, further enhancements are needed. This challenge might be addressed by building upon AlphaFold or integrating it with geometric deep learning (124).

Notably, AlphaFold’s prowess lies in its ability to deduce a protein’s 3D configuration from its primary amino acid sequence. From this, AlphaFold’s EvoFormer module learns complex patterns of AA interactions and predicts the distances and orientations of those interactions in 3D space, with the goal of essentially providing an estimated structural representation. Moreover, it uses a structure-based method for refining the coordinates of all heavy atoms within a protein (101). Because AlphaFold can generate detailed structural predictions from primary sequence information alone, its use in identifying relevant TCR-pMHC interactions is particularly intriguing. A recent approach utilizes a modified version of AlphaFold to resolve correct and incorrect peptide epitopes in TCR-pMHC interactions (125). This study suggested that supervision is required for appropriately applying the AlphaFold approach to TCR-pMHC systems: In comparison to the default AlphaFold (126), AlphaFold-Multimer (99), designed specifically to interrogate protein-protein structural complexes, more capably predicts TCR-pMHC binding specificity at a lower computational cost and higher accuracy.

## 4 Deep learning approaches

Deep learning, a machine learning subclass, is dramatically transforming the exploration and comprehension of TCR specificity. Machine learning excels in pattern recognition and prediction, making it versatile in applications like predicting cell types or antibody affinity based on gene expression profiles. However, the laborious feature extraction process, particularly with vast, feature-rich data, is a limitation. Deep learning alleviates this with an automated approach for feature extraction. Its layered structure facilitates capturing complex, high-dimensional data patterns, despite its interpretability challenges. CNN and RNN, two key Deep learning models, find varied biological applications, from image processing to protein engineering. Deep learning is poised to revolutionize TCR specificity understanding, and possesses the potential for ushering in the design of optimized immune treatment strategies.

### 4.1 Deep learning architecture

In contrast with the computational approaches discussed in detail thus far, which use physical equations and modeling to predict data, machine learning algorithms infer a relationship between inputs and outputs by learning from a set of hypotheses. This can be described by a collection of K training samples that may contain features x in an input space X (e.g. AA sequences), and corresponding labels y in output spaces Y (e.g. pairwise residue distances), where ${\left\{x_{j},y_{j}\right\}}_{j=1}^{N}$ are sampled independently and identically (i.i.d) from some joint distribution. Additionally, an identified function f:X→Y maps inputs to labels, and a corresponding loss function l:Y×Y→ℝ measures how far f(x) deviates from its corresponding label y . In supervised learning, the goal is to find a function f that minimizes the expected loss, _(*x*,*y*)~__*p*_[*l*(*f*(*x*),*y*)], for (x,y) jointly sampled from. Parameterization of the hypothesis class depends on the allowable choice of the network f in some allowable space ℱ .

**Figure 3:**
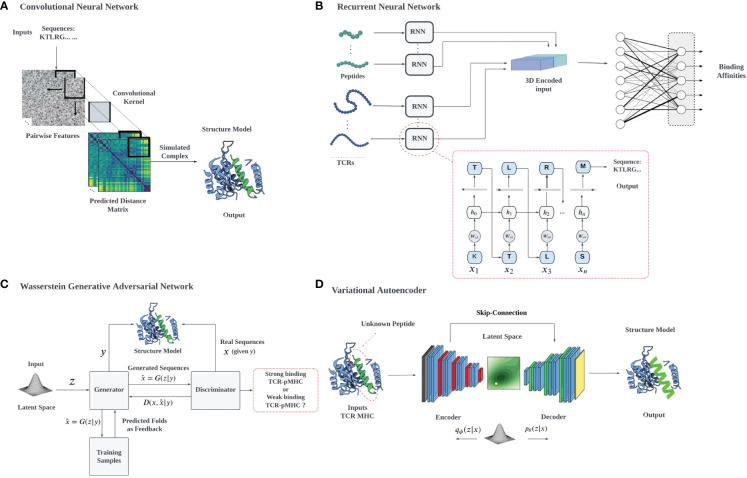
A schematic illustration of various deep learning architectures employed for TCR-pMHC interaction prediction: **(A)** 2D CNN-based prediction of TCR-pMHC interactions: The pairwise features of protein sequences are encapsulated in a 2D matrix representation, which serves as input for the 2D CNN. The CNN systematically samples the entire protein pairwise feature space, processing the data to facilitate the learning of TCR-pMHC interactions, **(B)** RNNs utilize auto-regressive learning to generate sequences, which can be applied in the context of TCR-pMHC interaction prediction, **(C)** In the GAN framework, a mapping from a prior distribution to the design space can be obtained through adversarial training, enabling the generation of novel TCR-pMHC interaction predictions, **(D)** VAEs can be jointly trained on protein sequences and their properties to construct a latent space that correlates with the properties of interest, for example, the TCR binding capacity of unevaluated target peptides.

Data analysis and deep learning predictions often overcome the traditional challenges of feature extraction in ML by recognizing relevant features, constructing hierarchical representations, handling large datasets effectively, providing end-to-end learning, and facilitating transfer learning, overcoming the limitations of classical approaches. High-dimensional data tasks can be efficiently handled with deep learning algorithms using hierarchical artificial neural networks. However, the interpretability of neural networks and deep learning can be a problem, due to their complexity, non-linearity, and the lack of physical interpretation and transparency due to their black-box nature. We will describe in detail the use of several common architectures (**Figure 3**), such as CNNs, RNNs, VAEs, and Generative Adversarial Networks (GANs), which have been developed for different applications, including biological problems such as cancer immunology (127).

#### 4.1.1 Convolutional neural networks

CNNs are a subtype of deep learning network architecture that have historically performed well on two-dimensional data with grid-like topologies, including images, and this approach is also applicable to other problems requiring shift-invariance or covariance (128). In order to capture this translational invariance, CNNs use convolutional kernels (feature extraction) for layer-wise affine transformations. There are three factors involved in the learning process of a CNN: sparse interaction, parameter sharing, and equivariant representation (129). CNNs utilize convolutional layers for sparse interaction, enabling efficient processing of high-dimensional data while reducing computational demands. Parameter sharing across input data locations decreases required parameters, enhancing training and inference efficiency. Lastly, equivariant representation ensures the network’s output remains invariant to input transformations, promoting generalization across diverse input variations. CNN has been applied to predict protein residue distance maps based on AA sequences (130) (**Figure 3A**). Convolutional operation ∗ with respect to the Kernel *W* and 2D data *X* (in this case, represented by residue-residue distance maps from AA sequences) can be expressed as


(3) $$\left(X*W\right)\left(i,j\right)=\sum_{m}\sum_{n}X\left(m,n\right)W\left(i-m,j-n\right),$$

Where (*X* ∗ *W*)(*i,j*) denotes the convolution output at position (*i,j*), and *X*(*m,n*) and *W*(*i* − *m,j* − *n*) represent the value of the input *X* at position (*m,n*) and the parameter of the kernel at position (*i*−*m,j*−*n*), respectively. One important variation on this general scheme that is relevant to the TCR-pMHC problem, called Residual Network (ResNet) (131), includes skip-connections between layers to recover spatial information lost during down-sampling. AlphaFold is one example of such an approach that uses ResNets to predict inter-residue distance maps of primary AA sequences (132).

CNNs can also be used to treat 3D protein structure prediction as a computer vision problem by voxelizing a given structure. One example is DeepSite (133), which uses voxelized representations of different atom types and deep CNNs to predict binding sites. Despite DeepSite’s potential to capture more interactions using voxelized representations and larger datasets, its performance appears lower than an alternative, template-free machine learning method (P2Rank) that applies clustering to score regions of a protein’s solvent accessible surface to identify candidate binding pockets (134). This discrepancy is possibly due to the CNN approach requiring even larger training dataset or differences in training set distributions. Yet another method employs a CNN-based segmentation model inspired by U-Net to predict binding sites in a single step (135). In general, U-Net is a CNN architecture originally designed to segment biomedical images. It utilizes symmetric encoder-decoder structures with skip connections between mirrored layers in both encoding and decoding paths, which allows accurate localization and the preservation of detailed information. In this method, a three-dimensional grid is generated around the protein, and each voxel within the grid is assigned a probability of being part of a binding pocket. The U-Net-inspired approach offers a more streamlined prediction process compared to traditional methods and has shown improved performance in detecting binding when compared to DeepSite, another prominent tool in the field. Overall, both P2Rank and U-Net-inspired methods offer unique advantages for the identification and prediction of protein-ligand binding sites.

#### 4.1.2 Recurrent neural networks

RNNs are neural networks that operate on sequential data (96), such as time series data, written text (i.e., NLP), and AA sequences (**Figure 3B**). The RNN algorithm can be represented by in the following mathematical setup, where a hidden state *h*^(^*^n^*^)^ is recursively solved using an initial value *h*^(0)^ and sequential data [*x*^(1)^*,x*^(2)^*,…,x*^(^*^N^*^)^], via


$$h^{\left(n\right)}=z^{\left(n\right)}\left(x^{\left(n\right)},x^{\left(n-1\right)},\ldots ,x^{\left(2\right)},x^{\left(1\right)}\right)=g\left(h^{\left(n-1\right)},x^{\left(n\right)};\theta \right).$$

Here, *θ* represents the RNN parameters, which include the weights and biases associated with the network’s connections, learned during the training process. The function *g* represents the update function describing the transformation from one position to another and utilizes the previous hidden state *h*^(^*^n^*^−1)^, current input *x*^(^*^n^*^)^, and *θ* parameters to produce the updated hidden state *h*^(^*^n^*^)^. *z*^(^*^n^*^)^ represents the cumulative transformation for position *n*. The hidden state vector contains all previously observed information at position *i*. Using this approach, sequential data of variable length can be fed to an RNN. This approach can be susceptible to a vanishing gradient, complicating optimization, and the ‘explosion problem’ (the error signal decreases or increases exponentially during training), potentially affecting the predictive accuracy and robustness of TCR-pMHC model. Specifically, the recurrence relation *h*^(^*^n^*^)^ = *g*(*h*^(^*^n^*^−1)^*,x*^(^*^n^*^)^;*θ*) in this context becomes especially vulnerable. When back-propagating through time over multiple steps, the gradient with respect to the loss function *L*, which measures the discrepancy between predicted and actual outcomes, can either shrink or grow exponentially. This behavior is due to the repeated multiplication by the weight matrix, as described by *∂L/∂h*^(^*^n^*^−^*^t^*^)^. If the network’s weights, in the context of TCR-pMHC modeling, are not properly initialized or regularized, it can lead to gradients significantly diverging from the ideal range. Consequently, LSTM networks (60), which are commonly used to mitigate this (136). An example of an LSTM approach in the context of specific TCR-Peptide binding prediction is using embedding vectors of AAs to construct a single vector, which can then be used as an LSTM (137) to learn long-range interactions within AA sequences; however, their efficacy depends on the formulation of the problem, the dataset characteristics, and the network architecture. In some situations, alternative deep learning approaches, such as CNNs and transformers, may be more applicable.

As an alternative to the recurrent network architecture, the attention mechanism is a method that can be used to improve the information processing ability of the neural networks (100). This mechanism is inspired by human biological systems that process large amounts of information by focusing on distinct parts and works by preventing the system from processing available information simultaneously (62). Attention-based models have several advantages over RNN models, including their parallelizability and ability to capture long-range relationships. The transformer model (62), a groundbreaking deep learning architecture is characterized by its self-attention mechanism, which enables the processing of input sequences in parallel rather than sequentially, distinguishing it from traditional attention mechanisms that typically rely on recurrent or convolutional layers. AlphaFold-Multimer (99) is one example of a transformer model that employs the attention-based model to generate models of TCR-pMHC interaction, which can then be used to distinguish correct peptide epitopes from incorrect ones with substantial accuracy. In directing these approaches to TCR-pMHC data in the future, these methods could be particularly helpful for predicting a target residue or the desired residue-specific properties of a target residue from the AA sequence of a protein. For example, transformer-based models have already been used to generate protein sequences conditioned on target structure and learn protein sequence data to predict protein-protein binding interfaces (138).

#### 4.1.3 Variational autoencoder

The AE neural network is an unsupervised learning algorithm based on backpropagation that sets its target values equal to the input values (61). This is typically accomplished by mapping input to latent space in the encoder and reverse mapping in the decoder (**Figure 3D**). The latent space’s dimension is less than the dimension of the original input and is constrained in some way (for example, by sparsity). In this framework, one assumes a set of unlabeled training vectors, {*x*^(1)^*,x*^(2)^*,x*^(3)^*,…*}, where *x*^(^*^i^*^)^ ∈ *^n^*. AE attempts to approximate the identity function in order to produce output *y* that is similar to *x* with respect to a loss function *L*: *n*


(4) $$\theta =\underset{y^{\left(i\right)}\in R^{n}}{\arg\min}\frac{1}{n}\sum_{i=1}^{n}L\left(x^{\left(i\right)},y^{\left(i\right)}\right)$$

In one AE application directed at TCR-pMHC interaction prediction (139), researchers predicted PPIs from AA sequences in order to identify key antigenic features to gain a more detailed understanding of the underlying immune recognition process.

VAEs (102) build on AEs by providing a stochastic mapping between the input space and a lower dimensional latent space, which is particularly useful when the input space follows a complex distribution. The latent space distribution typically takes a much simpler functional form, such as a multivariate Gaussian. Variational Inference (VI) (140) is a machine learning technique used in VAEs that approximates complex probability densities through optimization, allowing for efficient learning and data compression in the transformed latent space. Comparatively, it is faster than classical methods, such as Markov chains and MC sampling. In the VI method, the stochastic encoder is trained so that it approximates the true posterior distribution *p_θ_* (*z*|*x*) of the representation *z* given the data *x* with parameters *θ*, by means of the inference model *q_ϕ_* (*z*|*x*) with parameters *ϕ*, and weights parameterized by the data. In contrast, a decoder gives an estimate of the data given the representation, *p_θ_* (*x*|*z*). However, direct optimization is not computable; thus, training is done by maximizing the evidence lower bound (ELBO), Λ*_θ,ϕ_* (*x*), which gives a lower bound on the log-likelihood of the data:


(5) $${\text{Λ}}_{\theta ,\phi }\left(x\right)=E_{z∼q_{\phi }(z|x)}\text{log }p_{\theta }(x|z)-D_{KL}(q_{\phi }(z|x)||p_{\theta }(z|x))$$

where in general $E_{z∼q_{\phi }(z|x)}log\;p_{\theta }\left(z|x\right)$ represents the expected value of a function $log\;p_{\theta }\left(z|x\right)$ with respect to the conditional distribution $q_{\phi }(z|x)$ , which measures the average value of the function $log\;p_{\theta }\left(z|x\right)$ when considering all possible values of z , weighted by the probabilities assigned to them via $q_{\phi }(z|x)$ . $D_{KL}(q_{\phi }||p_{\theta })$ is the Kullback-Leibler divergence quantifying the distance between two distributions $q_{\phi }$ and $p_{\theta }$ , which represents the similarity of the latent space distribution with the target distribution p(z) . An example of VAE prediction in the TCR-pMHC interaction prediction field includes the CASTELO approach, which was used in combination with MD simulations to identify mutated versions of a known WT peptide that lead to enhancements in TCR-pMHC binding (103). Future applications of VAE-based prediction schemes will likely make an impact on describing TCR-pMHC interactions in combination with other preexisting strategies.

#### 4.1.4 Generative adversarial networks

GANs (141) are an emerging technique for both semi-supervised and unsupervised learning (142) that provide a method to obtain deep representations without the necessity to employ extensive training data annotations. In contrast to VAEs, GANs are trained through adversarial games between two models or networks (**Figure 3**): a generator network, G , which maps from latent space ${\mathbb{R}}^{\left|z\right|}$ of dimension |z| , to the space of data, $G:G\left(z\right)\to {\mathbb{R}}^{\left|x\right|}$ , where $z\in {\mathbb{R}}^{\left|z\right|}$ is a sample from latent space or simple distribution $p_{z}\left(z\right)$ (e.g. Gaussian), $x\in {\mathbb{R}}^{\left|x\right|}$ is a data-point, D is a discriminator function that maps an example to the probability that the example belongs to the real data distribution rather than the generator distribution (fake data), D:D(x)→(0,1) . This game-based setup trains the generator model G∈G by maximizing the error rate of the discriminator, D , so that the discriminator is fooled. On the other hand, the discriminator D∈D is trained to recognize fooling attempts. It is expressed as the following objective (143):


(6) $$\begin{array}{l} \underset{G\in G}{\min\;}\underset{D\in D}{\max}V\left(D,G\right)=E_{x∼p_{data}\left(x\right)}\left[\text{log }D\left(x\right)\right]+E_{z∼p_{z}\left(z\right)}\left[\text{log }(1-D\left(G\left(z\right)\right))\right]. \end{array}$$

In training, this loss function is optimized stochastically. Both the generator and discriminator are trainable via Standard Gradient Descent (SGD) algorithms. The discriminator can be updated *M* times for every generator update. After training, synthetic data is created using only the generator network.

GANs have been making rapid progress in continuous domains, but mode collapse and instabilities can occur when training this GAN objective and has made analyzing discrete sequences a significant challenge. One variation, referred to as the Wasserstein GAN (WGAN) (144, 145), introduces a penalty to constrain the gradients of the discriminator’s output, resulting in a more stable and trainable model. While GANs utilize a sigmoid function in the last layer for binary classification, the WGAN approach removes this function to approximate the Wasserstein distance (146), using Lipschitz discriminators: namely, that for discriminator function D there exists a constant L such that |D(x)−D(y)|≤L||x−y|| for any two points x and y in the input space. This ensures that the gradient of the discriminator’s output with respect to its input is bounded by some constant K:‖∇(D(x))‖≤K .

GANs can be used in protein modeling to produce new protein-like folds by learning the distribution of protein backbone distances. In one application, one network, *G*, generates folds, while a second network, *D*, distinguishes generated folds from fake ones (147). While WGAN models have not yet been widely applied to study TCR-pMHC specificity, they have been used to generate genomic sequence data (148). While their optimization behavior is generally well behaved, WGANs can exhibit undesired behavior in some applications. For example, in generating sequences containing particular motifs in the above application, in some cases, a strong motif match appeared twice in the same generated sequence because the final predictor score was insensitive to the presence of two motifs (the best match is used). Biologically, such sequences can be undesirable. Other technical issues that impact GAN approaches include unstable objective functions, mode collapse, variable length structure generation, conditioning difficulty, and the need to sample from a distribution instead of predicting a single output (149), and various approaches (144, 145, 150–153) have attempted to address these issues. GANs have influenced the field of sequence design, both when conditioning structural information (154) and when not (155, 156).

Diffusion models, an alternative to GANs, address many of these issues. Diffusion models are a class of latent variable models modeling the data generation process as iterative denoising of a random prior. They use a specific parameterization of the approximate posterior distribution that can be interpreted as an unobserved fixed prior diffusing to the observed posterior distribution (157). The diffusion model addresses some limitations of GANs by enabling explicit density estimation, reducing the mode collapse problem often seen in GANs, and providing more stable training procedures.

Due to several key differences, data-driven generative modeling methods have not had the same impact in the protein modeling setting as in the image generation setting. The first difference between proteins and images is that proteins cannot be represented on a discretized grid that is amenable to the straightforward application of generative models. Inconsistencies in the predictions of the pairwise distance matrix of a protein’s atoms lead to nontrivial errors when optimization routines are used to recover the final 3D structure when using existing models (158). Furthermore, proteins are not naturally oriented in a canonical manner like images. Therefore, rotationally invariant methods must account directly for this factor of variation in model weights. This reduces the amount of effective model capacity that can be dedicated to structural variation.

## 5 Hybrid approaches

In modeling natural systems, the exponential family of pairwise models is an important class of distributions to consider, which enjoys mathematically interpretable forms and is sufficiently general to include many of the common distributions, such as Gaussian, Poisson, and Bernoulli distributions (159). Additionally, pairwise models are commonly used in the statistical physics community for the analysis of categorical sequence data. There have been many successful applications of pairwise models such as the Ising model (160) or the generalized Potts model (91). One of the open questions in this area is how to train such models when additional higher-order interactions are present in the data that cannot be included in a pairwise model. Hybrid models addressed these issues, which combine a pairwise model with a neural network and can significantly improve pairwise interaction reconstruction. These hybrid approaches can often demonstrate performance improvements over alternative methods. We will focus on one particular example of a hybrid model recently developed to characterize systems-level TCR-pMHC specificity.

The Rapid Coarse-Grained Epitope TCR (RACER) (161, 162) model utilizes high-throughput TCR and peptide data, crystal structures, and a pairwise energy model to accurately predict TCR-peptide binding affinities. In this approach, supervised machine learning is applied to pre-identified TCR-peptide structures (45, 137) and experimental data to derive a coarse-grained, chemically accurate energy model of the TCR-pMHC interaction. While deep learning algorithms can implicitly capture higher-order interactions, they may still be limited by the availability of sequences. To mitigate this, RACER uses pairwise potentials to reduce the requirement for extensive sequence data. The optimization framework employed by RACER utilizes the AWSEM force field (163) to represent direct PPIs:


(7) $$V_{direct}=\sum_{\begin{array}{l} i\in \text{TCR}j\in \text{peptide} \end{array}}\gamma (a_{i},a_{j}){\text{Θ}}_{ij}^{I}$$

Where $\gamma_{ij}\left(a_{i},a_{j}\right)$ denotes the pairwise interaction between one of 20 AA residues $a_{i}$ and $a_{j}$ at positions i and j in the index TCR and peptide, respectively. ${\text{Θ}}_{ij}^{I}$ describes a sigmoidally decreasing ‘switching function’ that inversely weights each pairwise interaction based on inter-residue distance. In this model, TCR-peptide accurately assessed in a computationally efficient manner across entire immune repertoires using supervised machine learning to differentiate strong and weak binding pairs, assisting in identifying T cells specific to tumor antigens and enhancing cancer immunotherapy. Of course, the compromise for requiring fewer training sequences is the added requirement of a reasonable structure for the system of interest.

As we mentioned in Section 4, AlphaFold-Multimer (99), developed by DeepMind, can also be categorized as a hybrid model since this approach uses both sequence and structural information in training and predicting steps. AlphaFold-Multimer algorithm consists of two key processing elements, the input derived from MSAs and the evaluation of interatomic distances between AAs within a protein complex structure. A distance matrix provides spatial information for each AA pair, while the MSA aspect preserves and analyzes AA conservation and covariant properties. AlphaFold-Multimer uses the attention-based model to generate models of TCR-pMHC interaction that can be used to distinguish correct peptide epitopes from incorrect ones with substantial accuracy (164). In the future, AlphaFold’s ability to predict a collection of key structures could significantly enhance the predictive power of other hybrid approaches that rely on structural templates like RACER.

## 6 Discussion

This review has presented an overview of recent efforts to predict TCR-pMHC using theoretical, computational, and deep learning approaches, emphasizing both their strengths and limitations. We have explored sequence-based, structure-based, and hybrid methodologies for predicting TCR-pMHC interactions across species, emphasizing the growing importance of these computational techniques within the field. Predicting TCR-pMHC interactions based on AA sequences offers a number of advantages, including leveraging an abundance of publicly available data and using deep learning to extract meaningful features. This representation, however, is also inherently sparse and sample-inefficient, posing challenges. A traditional method of representing AA sequences often fails to encapsulate all essential information, despite the possibility of adding physical descriptors and biological characteristics.

Structure-based models incorporate 3D information crucial for binding and signaling. Nonetheless, challenges arise from the complexity of raw 3D data and the high interdependence of variables within the structure. While graph-based and surface-based representations via Graph Neural Networks and geometric deep learning frameworks have shown promise, they require meticulous model design and implementation, and the invertibility of 2D projections to the original 3D structure is not guaranteed. Hybrid models, combining pairwise models with neural networks, effectively address the issue of higher-order interactions unaccounted for in traditional pairwise models, leading to improved performance in reconstructing pairwise interactions. Hybrid models, despite their ability to handle higher-order interactions, are limited by the requirement for well-defined system structures and extensive sequence data, and their complexity may hinder interpretability and computational efficiency.

With respect to understanding TCR-pMHC specificity, Recent modeling approaches commonly integrate deep neural network techniques with more traditional methods like cluster analysis. To-date, successful models of TCR-pMHC interactions attempt to deliver on a subset of important objectives, including 1) Computationally efficient characterization for large-scale implementation, 2) Sensitivity in recognizing novel favorable TCR-pMHC pairs, 3) Specificity in predictions through demonstrating the identification of non-recognition pairs, 4) Accurate predictions on data that are far away from the training data, including completely new test TCRs or peptide and 5) Accurate predictions on exhaustive test data that is very close to training examples, including the classification of all point-mutations of a previously identified peptide. At present, no current model adequately addresses all of these objectives. Because of the sheer allowable diversity of TCR and peptide feature space, sparsity in available training data will be a persistent challenge in future applications.

Because of the significant clinical implications of successful models of TCR-pMHC specificity, the number of newly developed approaches is rapidly expanding. As a result, we advocate for standardization in the testing protocols. Because new models are often trained on data that is distinct from that of other previous models, comparative performance is often highly sensitive to the choice of test data. This can artificially enhance the perceived predictive utility of a new model or unreasonably diminish the ability of existing models. Comparative predictive assessments, when performed, should utilize data with neutral similarity to either model. Despite considerable progress in this domain, numerous challenges and future research directions remain. To gain valuable biological insights from TCR-pMHC binding prediction models, current limitations must be addressed and their generalizability, interpretability, and precision must be improved. Enhancing precision involves integrating diverse data modalities and high-quality sources, with special attention given to those reflecting epitope mutations. Improving generalizability entails training models on comprehensive datasets that span both known and novel epitopes, ensuring robustness across varied biological conditions. Crucially, models must be interpreted in a way that translates complex computational outputs into biologically meaningful insights, advancing our understanding of immune responses beyond mere computational contexts. Such targeted improvements will catalyze the development of potent and precise immunotherapies. TCR-pMHC interactions are expected to benefit substantially from new advances in data availability and computational techniques as the availability of high-quality data increases. As a result, innovative therapeutic approaches and tailored medical treatments will be developed based on a deeper understanding of their functions in human health and disease.

In the exhaustive analysis of various methodologies for inferring TCR specificity, our study finds no single superior approach. Rather, we propose a dynamic, integrated strategy that transcends traditional methods and embraces a confluence of techniques while remaining receptive to continual advancements. This multifaceted approach emphasizes the importance of harnessing unlabelled TCR sequences and leveraging data augmentation techniques. It also calls for the integration of both sequence- and structure-aware features, coupled with the application of cutting-edge computational techniques. Furthermore, we underscore the critical need for a collaborative ecosystem that fosters interactions among experts from disparate domains, including immunology, machine learning, and both translational and industrial sectors. Such synergy is pivotal in driving forward-thinking solutions, and we advocate for the unobstructed accessibility of successful models to promote open collaboration and accelerate progress in TCR specificity prediction.

## Author contributions

JG supervised the work, ZG performed the review, ZG and JG wrote the paper. All authors contributed to the article and approved the submitted version.
